# Modern Ubasute: Public Discourse and Sentiment about Older Adults and COVID19 Using Machine Learning

**DOI:** 10.1093/geroni/igaa057.3485

**Published:** 2020-12-16

**Authors:** Xiaoling Xiang, Xuan Lu, Alex Halavanau, Jia Xue, Yihang Sun, Patrick Ho Lam Lai, Zhenke Wu

**Affiliations:** 1 University of Michigan, Ann Arbor, Michigan, United States; 2 Stanford University, Menlo Park, California, United States; 3 University of Toronto, Toronto, Canada; 4 University of Michigan-Ann Arbor, Ypsilanti, Michigan, United States

## Abstract

This study examined public discourse and sentiment on social media regarding older adults in COVID-19. Twitter data (N=82,893) related to both older adults and COVID-19 and dated from January 23rd to May 20th, 2020, were analyzed. Classification of tweets involved supervised machine learning. Latent Dirichlet Allocation was used to identify dominant themes in public discourse using, accompanied by a qualitative thematic analysis. Sentiment analysis was conducted based on the NRC Emotion Lexicon. The most common category in the coded tweets was “personal opinions” (66.2%), followed by “informative” (24.7%), “jokes/ridicule” (4.8%), and “personal experiences” (4.3%). More than one in ten (11.5%) tweets implied that the life of older adults is less valuable or downplayed the pandemic because it mostly harms older adults. A small proportion (4.6%) explicitly supported the idea of just isolating older adults. Almost three-quarters (72.9%) within “jokes/ridicule” targeted older adults, half of which were “death jokes.” The daily average of ageist content was 18%, with the highest of 52.8% on March 11th, 2020. We extracted 14 themes, such as perceptions of lockdown and risk. A bivariate Granger causality test suggested that informative tweets regarding at-risk populations increased the prevalence of tweets that downplayed the pandemic. The COVID-19 pandemic has exposed and intensified ageism in our society. Information about COVID-19 on Twitter influenced public perceptions of risk and acceptable ways of controlling the pandemic. Public education on the risk of severe illness is needed to correct misperceptions.

